# Advance of autophagy in chronic kidney diseases

**DOI:** 10.1080/0886022X.2016.1274662

**Published:** 2017-01-13

**Authors:** Xu Deng, Yifan Xie, Aihua Zhang

**Affiliations:** a Department of Nephrology, Children's Hospital of Nanjing Medical University, Nanjing, China;; b Jiangsu Key Laboratory of Pediatrics, Nanjing Medical University, Nanjing, China

**Keywords:** Autophagy, macroautophagy, microautophagy, chaperone-mediated autophagy, chronic kidney diseases

## Abstract

Autophagy, a highly conserved mechanism for cell survival, emerges as an important pathway in many biological processes and diseases conditions. Studies of cultured renal cells, human kidney tissues and experimental animal models implicate that autophagy regulation is the critical aspects in chronic kidney diseases (CKD). Here, we summarize the current studies on the role of autophagy in CKD. Unveiling the precise regulation mechanism of autophagy in CKD is essential for developing potential prevention, diagnostic and therapeutic targets of these sticky clinical challenges.

## 1. Autophagy

Autophagy is a tightly precise system in which the endogenous aggregates cellular protein and damaged organelles are removed via the lysosomal pathway. Except for eliminating materials, autophagy also serves as a highly efficient recycling system that produces new components and energy for cellular renovation and balance of homeostasis. Depend on different delivering mechanism of cargo to the lysosome, autophagy is classified at least into three types: microautophagy, macroautophagy, and chaperone-mediated autophagy (CMA).

### 1.1. Macroautophagy

Evolutionarily-conserved macroautophagy activated in response to stressful condition is a lysosomal-mediated intracellular degradation process.^1^^,^^2^ The process starts with the formation of a structure on the endoplasmic reticulum (ER), called the omegasome. Then, it expands into a phagophore or isolation membrane to assemble into the precursor of the autophagosome. During phagophore elongation, cytoplasmic components are assembled within this structure. Next, it forms a closed double-membrane vesicle, called an autophagosome. Last, amphisomes fuses with acidic lysosomes, forming autolysosomes, where the entrapped contents are degraded.^3^ This process is not only the fundamental in disposing of aged and aggregated proteins, but also is the known mechanism for ridding of damaged organelles under normal conditions as well as stress conditions.^4–8^ In states of normal physiology, there is an active autophagic process working in all cell types of the kidney and is especially prominent in podocytes to protect cellular integrity against the stresses, which these cells encounter at the filtration barrier.^9^ Meanwhile, autophagy is also participated in various pathological processes of renal diseases. What’s more, macroautophagy level declines with age, leading to cellular age-related waste accumulation, a cause of the progression of ageing process.^10^

### 1.2. Microautophagy

Microautophagy is a nonselective type of autophagy. It involves engulfment of cytoplasmic cargo by direct invagination of the lysosomal membranes into autophagic tubes, which mediate both invagination and vesicle scission into the lumen. Although microautophagy is constitutive, it can be induced by starvation and rapamycin.^11^ Microautophagic invagination and budding regulate lysosomal size by consuming superabundant membrane formed during macroautophagy.^12^ However, the exact functions of microautophagy in mammalian cells are not yet fully understood.^13^ It has been suggested that microautophagy is functions in the maintenance of organellar size, membrane composition, cell survival under nitrogen restriction, and the transition pathway from starvation-induced growth arrest to logarithmic growth.^14^

### 1.3. Chaperone-mediated autophagy

CMA is a type of selective autophagy, so far only identified in mammalian cells. Different from other types of autophagy, its substrates, mainly cytosolic proteins, enter the lysosome.^15^ Proteins degraded by CMA are identified one-by-one by a cytosolic chaperone utilizing heat shock chaperone of 70 kDa (hsc70), which recognizes the pentapeptide KFERQ motif upon misfolding of protein complexes. This hsc70-protein complex binds to a lysosomal receptor complex and translocate into the lysosome.^16–18^ Thus, three events—(a) individual identification of single proteins, (b) unfolding before degradation, and (c) translocation inside the lysosomes—constitute the typical signature of CMA.

## 2. Types of autophagy involved in chronic kidney diseases (CKD)

The kidney is important for healthy living. It regulates the body fluids and blood pressure, excretes waste products and produces red blood cells.^19^ Human kidney receives nearly 25% of cardiac output and consumes 7% of daily energy expenditure.^20^ CKD, characterized by a glomerular filtration rate below 60 mL per minute for over 3 months,^21^ is the end stage of various kidney diseases, leading to poor health outcomes and very high health care costs. Fibrosis is responsible for chronic progressive kidney failure, and the prevalence of CKD is increasing worldwide.^22^^,^^23^ Other mechanisms, such as oxidative stress,^24^ inflammation,^25^^,^^26^ mitochondrial damage,^27^^,^^28^ ER stress,^29–31^ and autophagy,^32^^,^^33^ are also involved in the progress of pathophysiology of CKD.

Autophagy is associated with the pathogenesis of a number of diseases. However, little is known about the relationship between autophagy and kidney diseases, especially autophagy and CKD. Until currently, emerging body of evidences suggest that autophagy plays a crucial role in chronic kidney diseases.

### 2.1. Macroautophagy and CKD

Diabetic nephropathy (DN) refers to CKD initiated by diabetes mellitus. There is a growing evidence that autophagy or mitophagy is altered in DN. An early study in diabetic rats detected an association between decreased autophagic activity and densities of autophagic vacuoles in proximal tubular epithelial cells.^34^ Accordingly, a similar study suggested that regulation of blood glucose with insulin treatment led to the normalization of the density of autophagic vesicles, suggesting that this phenomenon was glucose-mediated.^35^

### 2.2. Endoplasmic reticulum autophagy and CKD

Autophagy eliminates ER filled with unfolded proteins, which is called ER-phagy. Bernales et al. discovered and first named “endoplasmic reticulum autophagy (ER-phagy)” during their unfolded protein response (UPR) study.^36^ This ER-phagy can maintain homeostasis of ER. ER-phagy seems to be cytoprotective against various folding diseases, including neurodegenerative diseases and diabetes.

ER is one of the multiple membrane sources for autophagosome biogenesis.^37^ During prolonged ER stress, nonselective macro-autophagy can be induced,^38^ and was suggested to serve as a backup for ER-associated degradation.^39^^,^^40^ Most notably, ER-phagy is also induced by ER stress,^36^ which does not require the macroautophagy machinery components.^41^ Thus, ER-phagy can maintain homeostasis of ER. ER-phagy seems to be cytoprotective against various folding diseases, including neurodegenerative diseases and diabetes.

### 2.3. CMA and CKD

In CMA, proteins containing KFERQ motif are selectively recognized by the cytosolic chaperone, hsc70. And in turn, the hsc70/substrate protein complex binds to the lysosomal membrane via lysosome-associated membrane protein 2A (LAMP-2A), leading to unfolding of the substrate and translocation inside the lysosome for subsequent degradation.^42^

Up to now, the role of CMA in the kidney has not been systematically studied. Sooparb et al. found that if CMA was suppressed, PAX2 expression increased in cultured cells. PAX2 was an important factor in kidney development, indicating the possibility that CMA and PAX2 could play roles in epithelial cell growth.^43^*In vivo*, Sooparb et al. reported the abundance of proteins in diabetic kidneys containing the CMA KFERQ signal motif and individual KFERQ-containing proteins (e.g., PKM2, GAPDH, and PAX2) were more abundant in diabetic rats. LAMP2A and HSPA8 decrease in cortical lysosomes from diabetic vs. control rats, indicating the possibility that CMA might be increased in diabetic kidneys.^43^

## 3. Autophagy in different cell types of kidney

### 3.1. Glomerular mesangial cells

Glomerular mesangial cells are specialized contractile pericytes, which locate in the centrilobular region called the mesangium and together with adjacent glomerular capillary endothelial cells and podocytes to regulate glomerular filtration. They are major targets of various glomerular diseases, such as IgA nephropathy and DN. In response to injury and progressive kidney diseases, mesangial cells proliferate and produce excessive extracellular matrix (ECM), leading to the development of glomerulosclerosis and kidney fibrosis, which is closely associated with the development and progression of CKD.

The role of autophagy in mesangial cells is not well recognized until recently. Wang et al. reported that autophagy was induced in mesangial cells following exposure to the heavy metal cadmium, an environmental toxin which accumulated in the kidneys and caused nephrotoxicity.^44^

Under the condition of serum deprivation, autophagy was induced by transforming growth factor-β1(TGF-β1) in mesangial cells, and autophagy-enhanced survival by preventing mesangial cells from undergoing apoptosis.^45^ The balance between synthesis and degradation of mesangial matrix is crucial progress in CKD. Studies unveiled a novel role of autophagy in negatively regulating the matrix production in mesangial cells by promoting the degradation of intracellular collagen I(Col-I), confirming that Col-I and aggregated, insoluble procollagen I undergo intracellular degradation through autophagy.^45^ Consistent with this, Kim et al. also found increase of Col-I deposition in the kidney in UUO model of Beclin 1± mice and mesangial cells with reduced expression of Beclin 1. Then, their further studies unveiled that the activators of autophagy reduces protein levels of Col-I induced by TGF-β1 in mesangial cells.^46^

### 3.2. Podocytes

Podocytes, highly differentiated epithelial cells lining the outer aspect of the glomerular basement membrane, is one of the important components of the filtration barrier. Podocyte injury is associated with marked proteinuria and decrease of glomerular filtration rate. Additionally, terminally differentiated podocytes are vulnerable to varity of injury, and the loss of podocytes is considered a key character of glomerular disease. Studies had confirmed that glomerular podocytes exhibited significant levels of autophagosomes even under basal conditions.^9^^,^^47^

Aldosterone (Aldo) is involved in the development and progression of CKD. Study found Aldo-induced podocyte apoptosis, autophagy, ROS generation, and downregulation of nephrin protein in a time-dependent manner.^27^ Accordingly, Yuan at al detected Aldo-induced ER stress and podocyte autophagy both *in vivo* and *in vitro*, and blockade of ER stress significantly reduced aldosterone-induced podocyte injury.^48^

Podocyte-specific deletion of the Atg5 gene resulted in proteinuria, loss of podocytes, and aging-related glomerulosclerosis indicating the critical importance of autophagy for glomerular maintenance.^9^ Autophagy-specific Atg5 or Atg7conditional knockout mice exhibited enhanced vacuolization in podocytes and tubularcells and ultimately resulted in FSGS and organ failure.^33^ A study comparing renal biopsies from patients with minimal change disease and patients with focal segmental glomerulosclerosis (FSGS), showed higher levels of Beclin 1-mediated autophagic activity in minimal change disease patients than those from FSGS patients, suggesting decreasing levels of autophagy might lead to progress to FSGS.^49^

### 3.3. Tubular epithelial cells

Renal tubular epithelial cells (RTECs) are key targets in acute kidney injury (AKI) and CKD, and histological studies have indicated that renal function correlates better with tubular and interstitial changes than glomerular changes. Renal tubulointerstitial fibrosis is the hallmark of progressive CKD and end-stage renal diseases (ESRD) accompanied by tubular degeneration and atrophy.

Cyclosporine is a potent immunosuppressive drug widely used in preventing transplant rejection and treating autoimmune diseases, but its long-term use leads to the development of a chronic nephrotoxicity characterized by tubular atrophy, interstitial fibrosis, glomerulosclerosis, and impaired renal function. Cyclosporine induces ER stress, which is a potent stimulus for autophagy activation. Autophagy inhibition during cyclosporine treatment with beclin1 siRNA significantly increases tubular cell death.^50^ Thus, induction of autophagy is protective against cyclosporine-induced tubular cell death.^50^

TGF-β1 is known as the most potent profibrotic cytokine known. Studies in cultured human renal proximal tubular epithelial cells demonstrated that TGF-β1 induced upregulation of autophagy-related genes, *Atg5*, *Atg7*, and *Beclin 1*, and accumulation of autophagosomes in a time- and dose-dependent manner.^51^ Furthermore, TGF-β1 activated autophagy through the generation of ROS, and promoted apoptosis in tubular cells.^51^

Induction of autophagy was reported in a transgenic mouse model with tertracycline-controlled overexpression of TGF-β1 in RTECs.^52^ The ureteral obstruction (UUO) model is a well-established model of progressive renal interstitial fibrosis. Increased autophagy with apoptosis and necrosis in tubules was also demonstrated in the renal fibrosis model, induced by UUO.^53^^,^^54^ What’s more, Yan Ding et al. found autophagy is induced primarily in RTECs of obstructed kidneys after UUO, and further study in UUO model of LC3 null (LC3^−/−^) mice and beclin 1± mice unveiled that deficiency of autophagic proteins lc3 and beclin1 results in increased collagen deposition in obstructed kidneys.^32^

### 3.4. Endothelial cells

The glomerular endothelial cells are important components of the glomerular filtration unit, together with the podocytes and mesangial cells to their contribution to glomerular barrier function. Renal endothelial dysfunction, therefore, leads to kidney dysfunction and may contribute to progression of CKD and renal fibrosis. In this regard, the renal endothelium has garnered much interest, however, its role in the initiation and the progression of renal fibrosis remains not well understood. Akin to EMT in tubular epithelial cells, the concept that endothelial cells may also acquire functional and structural characteristics of mesenchymal cells, called endothelial–mesenchymal transition (EndoMT), following tissue injury.^55^

EndMT plays a significant role in kidney fibrosis.^56^ Studies showed that hyperphosphatemia induced endothelial autophagy, possibly through the inhibition of the Akt/mTOR signaling pathway, which might play a protective role against high Pi-induced apoptosis in cultured human microvascular endothelial cell. What’s more, rat model of CKD renal tissue revealed that hyperphosphatemia was associated with increased endothelial LC3 staining.^57^

## 4. Autophagy pathways in CKD

Recent studies revealed dysregulated autophagy characterized by fibrosis in various tissues, including cardiac fibrosis, liver fibrosis, and idiopathic pulmonary fibrosis.^58^ Here, we review several autophagy signaling pathways in CKD.

### 4.1. Phosphoinositide 3-kinase/protein kinase B (PI3K/akt) signal pathway

Phosphoinositide 3-kinase/protein kinase B(PI3K/Akt) has vital significance in autophagy.^59^ DN is one of the major causes of ESRD, and the incidence of DN is increasing worldwide. Li et al demonstrated that high-glucose (HG) induced upregulation of (Pro)renin receptor played an important role in the reduction of autophagy and enhanced apoptosis in podocytes. And then, they further unveiled that the PI3K/Akt/mTOR/ULK1 signaling pathway mediated this effects.^60^ Consistent with their study, Lu et al found the mesangial cells exposed to HG showed upregulated miRNA-21 expression, downregulated PTEN protein and mRNA expression, upregulated p85PI3K, pAkt, pmTOR, p62/SQSTMI, and Col-I expression and downregulated LC3II expression. LY294002, inhibitor of PI3K, inhibited HG-induced mesangial cell hypertrophy and proliferation, downregulated p85PI3K, pAkt, pmTOR, p62/SQSTMI, and Col-I expression and upregulated LC3II expression.^61^

### 4.2. Rapamycin (mTOR) signal pathway

High evolutionarily conserved mechanistic target of rapamycin (mTOR) is a known pathway negatively regulates autophagy.^62^^,^^63^ Study suggested the induction of autophagy preceded tubular apoptosis and interstitial fibrosis and peaked after 3 days of UUO in the obstructed kidney of rats, and inhibition of autophagy with 3-MA enhanced tubular cell apoptosis and interstitial fibrosis.^64^ The expression of Akt and mTOR was decreased over the first 3 days in the obstructed kidney after UUO, implying that Akt-mTOR signaling might regulate activation of autophagy in the UUO model.^64^ These studies indicate that autophagy has a beneficial role in alleviating tubular damage and kidney fibrosis. Meanwhile, current study reported high Pi-induced autophagy activity in endothelial cells possibly through inhibiting the Akt/mTOR signaling pathway.^57^

### 4.3. TGF-β signal pathway

In progressive kidney diseases, fibrosis represents the common pathway to ESRD, regardless of the initial causes. TGF-β1 is the most potent profibrogenic factor for the development and progress of renal fibrosis.^65^ Studies showed that TGF-β1-activated autophagy via ROS, and autophagy promoted apoptosis in tubular cells.^51^ Ding et al. revealed that TGF-β1 upregulated LC3 through the Akt pathway.^45^ Kim et al. confirmed that TGF-β1-induced autophagy in mesangial cells by the TAK1-MKK3-p38 signaling pathway and concluded that the dual functions of TGF-β1, as both an inducer of Col-1 synthesis and an inducer of autophagy and Col-1 degradation underscore the pleiotropic function of TGF-β1.^46^ Report also found that the induction of autophagy in RTECs protected against apoptosis and promoted TGF-β1 degradation, thereby reducing TGF-β1 secretion could prevent the development of renal interstitial fibrosis.^32^

Induction of autophagy was reported in a transgenic mouse model with tertracycline-controlled overexpression of TGF-β1 in RTECs.^52^ Tubular overexpression of TGF-β1-induced dedifferentiation and decomposition of tubular cells by autophagy and tubule interstitial fibrosis, but not epithelial-to-mesenchymal transition, and no sign of apoptosis, implying that autophagy might mediate tubular cell death.^52^

## 5. The advantages and disadvantages of autophagy in CKD

The role of autophagy in progression of CKD is a controversial issue. On the one hand, autophagy may contribute to the degradation of Col-I and active TGF-β1 to inhibit kidney fibrosis. Wan-young Kim showed inhibition of autophagy by 3-MA enhanced tubular cell apoptosis and tubulointerstitial fibrosis in the obstructed kidney after UUO, implying that autophagy played a protective role in obstructed rat kidney.^64^ Study also showed that kidneys from mice deficient in autophagic protein Beclin 1 or knockdown by specific siRNA in primary mouse mesangial cells exhibited profibrotic phenotype with increased Col-I deposition, whereas treatment with inducers of autophagy resulted in decreased Col-I induced by TGF-β1 in primary mouse mesangial cells.^46^ According to these, LC3^−/−^ mice resulted in increased collagen deposition and increased mature profibrotic factor TGF-β1 levels in obstructed kidneys and Beclin 1± mice also displayed increased collagen deposition in the obstructed kidneys after UUO^32^. Genetic ablation of autophagy by proximal tubular epithelial cell-specific deletion of Atg5 also explored deposition of Col-I.^66^

On the other hand, autophagy has been suggested to induce tubular atrophy and decomposition to promote fibrosis. Some recent studies had consistently demonstrated that UUO-induced autophagy in proximal tubules (14 days of duration) may act in concert with apoptosis to induce tubular atrophy and nephron loss that was associated with the progression of interstitial fibrosis.^53^^,^^54^^,^^67^ Dong et al. found detected persistent autophagy in kidney proximal tubules of UUO mice. UUO-associated fibrosis—as indicated by the expression of ACTA2/a-smooth muscle actin, vimentin, fibronectin1, and collagen fibrils—was suppressed by pharmacological inhibitors of autophagy and also by kidney proximal tubule-specific knockout of autophagy-related 7 (PT-Atg7 KO) mice.^68^

## 6. Prospective

CKD is a complex pathological process. It is not only a decline of glomerular filtration rate, but also is accompanied by a variety of metabolic changes: abnormal lipid metabolism, calcium and phosphorus metabolism disorder, water and salt metabolism unbalance, reduction of erythropoietin production. Furthermore, CKD is the key to progress to ESRD, which can lead to distant organs damage, such as heart, lung, liver, and so on. Various metabolic factors and different organs influence each other, leading the injury of CKD to moving forward, finally resulting in high mortality. Therefore, study of the occurrence and development mechanism of CKD has a very important clinical significance to prevent and the treatment of ESRD.

Autophagy involves in the whole process of the development of CKD. But past studies mainly focused on macroautophagy, while mitoautophagy, lipidautophagy, ER-phagy, and CMA are largely studied. Recent studies show that plasmid metabolism, ER stress and UPR play a very important role in the pathogenesis of CKD. Therefore, future studies should use transgene technology to investigate other types of autophagy and their precise mechanism in CKD.

The kidney contains numerous types of local cells. Recent studies mainly pay attention to podocytes, tubular epithelial cells, and mesangial cells, other cell types remains unexplored. However, the kidney is consisted by glomerulus, which contains millions of vascular endothelial cells. Damage of endothelial cells has much to do with renal function. Current researches has found that autophagy in endothelial cells is closely related cardiovascular diseases. Just because of this, as a major component of the glomerular, autophagy in endothelial cells could possibly plays a critical role in the CKD, and more attention should be paid to this issue.

Every coin has two sides. The pros and cons of autophagy attract growing attention recently. Autophagy can act as a mechanism of cell survival, but it can also lead to cell death in response to persistent stress. Depending on the cell or tissue types and pathological settings, autophagy can be profibrotic or antifibrotic in different conditions. Meanwhile, the etiology of CKD is very complex, as well as its pathological changes. Therefore, to find the common aspects of autophagy in occurrence and development of CKD, and different aspects of autophagy in CKD has vital significant clinical importance.

Taken together, these studies implicate that autophagy plays a vital role in the development and progression in CKD, and unveiling the precise regulation mechanism that promises a new preventive therapeutic target to mitigate the pathogenesis of CKD.
